# Consequences of early postnatal benzodiazepines exposure in rats. I. Cognitive-like behavior

**DOI:** 10.3389/fnbeh.2014.00101

**Published:** 2014-03-28

**Authors:** Anna Mikulecká, Martin Šubrt, Aleš Stuchlík, Hana Kubová

**Affiliations:** Institute of Physiology, Academy of Sciences of the Czech RepublicPrague, Czech Republic

**Keywords:** benzodiazepines, clonazepam, cognitive functions, development, rats

## Abstract

Clinical and experimental studies suggest possible risks associated with the repeated administration of benzodiazepines (BZDs) during the prenatal or early postnatal period on further development and behavior. In the present study, we assess short- and long-term effects of early exposure to clonazepam (CZP) on cognitive tasks. CZP (0.5 or 1.0 mg/kg/day) was administered from postnatal day (P)7 until P11, and animals were exposed to the following behavioral tests at different developmental stages: (1) a homing response (HR) test, which exploits the motivation of a rat pup to reach its home nest, was administered on P12, P15, P18 and P23 rats; (2) passive avoidance was tested in three trials (at 0, 2 and 24 h intervals) on P12, P15, P18, P25 and P32 rats; (3) within- and between-session habituation was tested in an open field (OF) at P70; and (4) a long-term memory (LTM) version of the Morris water maze (MWM) was tested at P80. A 1.0 mg/kg dose of CZP extended latency in the HR and decreased the number of correct responses when tested at P12 and P23. In the first trial of the passive avoidance test, latency to enter a dark compartment was shorter in the CZP-exposed rats. Both treated and control animals older than P15 learned the passive-avoidance response at the same rate. Irrespective of the treatments, all adult animals showed within-session habituation. Between-session habituation, however, was found only in the controls. With respect to the MWM test, all animals learned to reach the platform, but animals exposed to higher doses of CZP spent more time swimming in the first acquisition test. No difference between groups was found in a repeated acquisition test (10 and 40 days after the first acquisition test). The results of the present study show that even short-term exposure to CZP alters behavioral responsiveness in pre-weaning, juvenile and adult animals. Not only were changes observed on conventional cognitive tests in our study, but the changes also seem to be related to emotional/motivational responsiveness.

## Introduction

Experiences during early life critically affect the development of the brain. The ultimate effect of early life experiences can contribute to either risk or resilience to neuropsychiatric conditions later in life. In rats, the 1st week of life represents a period of intense development of neural systems involved in the processing of non-spatial and spatial memory. Cognitive representation emerges and develops as rat pups first begin to explore their environment, and it continues to develop throughout adolescence (Ainge and Langston, 2012). Published data demonstrate that these hippocampal dependent functions are established between the 2nd and 3rd weeks of life (for review Avishai-Eliner et al., 2002).

Benzodiazepines (BZDs), which are psychoactive drugs commonly used among all age groups of patients, possess marked anxiolytic, sedative, hypnotic, and anticonvulsant properties. Preclinical and clinical studies suggest stable therapeutic effects of BZDs during development (Kubová and Mareš, 1989; Kubová et al., 1993). While cognitive impairment represents one of the most frequent behavioral alterations reported during acute treatment, such impairment usually disappears shortly after therapy withdrawal. Enduring cognitive alterations following the termination of exposure to BZDs is not documented in adult patients (for review Lader, 2011). Nevertheless, sparse developmental studies suggest that exposure of the immature brain to BZDs can result in cognitive alterations lasting long after the cessation of BZD exposure. However, results of these studies are inconsistent. Some studies have documented impairment in cognitive tests, whereas other studies find no difference between exposed animals and controls (for review, see Tucker, 1985). The results are often not directly comparable because of differences in the schedules of drug administration, different ages among those tested, and differences in tests. In addition, in most published studies, animals are exposed to BZDs through several developmental stages, beginning in the prenatal period. In many of these studies, exposure duration is extremely long, lasting through gestation and pre-weaning periods, thus making it impossible to specify a critical period of increased vulnerability of the immature brain to BZD-induced functional deficits later in life.

We designed a series of experiments to answer the following questions. Does short-term exposure to therapeutically relevant doses of clonazepam (CZP) during early postnatal development (1) lead to disturbances of cognitive-like behavior in immature and juvenile rats or (2) impair cognitive-like behavior later in adulthood? To address these questions, rat pups were injected intraperitoneally with CZP in doses of 0.5 and 1.0 mg/kg/day for 5 consecutive days starting at P7. The doses used are in the anticonvulsant dose range in rodent seizure models for these age groups of rats (Kubová and Mareš, 1989; Mikulecká et al., 2011). CZP was selected because it is a classic BZD with pronounced anticonvulsant and anxiolytic effects but with only mild sedative effects (Nardi and Perna, 2006). We used a HR test and a passive-avoidance test in the pre-weaning and juvenile periods, as well as the habituation of exploratory activity within an open-field (OF) arena and the long-term memory (LTM) version of the Morris water maze (MWM) in adults.

## Methods

### Animals

Male albino Wistar rats (Institute of Physiology, Academy of Sciences, Prague) were used (*n* = 160). Animals were maintained under controlled temperature (22 ± 1°C) and humidity (50–60%) with a 12/12 h light/dark cycle (lights on at 6:00 AM). Food and water were provided ad libitum (with the exception of the testing period). On day 5 (birth counted as day 0), the pups were randomly fostered, and each litter was adjusted to 10 males. The animals were weaned at postnatal day (P) 28. To exclude the participation of a litter effect, only a limited number of animals from the same litter were used. Experiments were approved by the Animal Care and Use Committee of the Institute of Physiology, Academy of Sciences of the Czech Republic, (v.v.i) and determined to be in agreement with the Animal Protection Law of the Czech Republic, which is fully compatible with the guidelines of the European Community Council directives 86/609/EEC.

### Drug exposure

CZP (Hoffmann - Switzerland; obtained as a gift) was suspended in one drop of Tween 80 and 1 ml of saline. Subsequently, this solution was diluted in such a way that all used doses of CZP were administered in the same volume of 5 μl/g of body weight. The doses were selected according to our previous studies on anticonvulsant effects of CZP in developing animals (Kubová and Mareš, 1989; Mikulecká et al., 2011). CZP at the doses of 0.5 and/or 1.0 mg/kg was administered intraperitoneally for 5 consecutive days from P7 to P11. Control siblings received the corresponding volume of vehicle. Animals were weighed daily during their exposure to CZP, and their overall health condition was regularly examined.

### Behavioral tests

**Figure 1:**
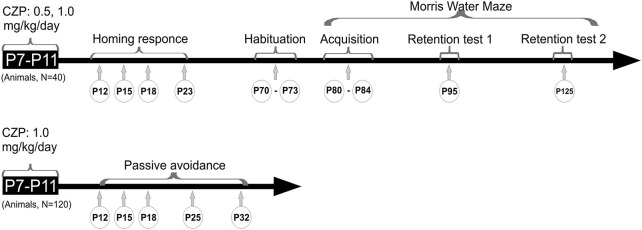
**Design/time diagram of CZP experimental procedure**. The upper part shows a timeline for behavioral tests such as homing response (HR), habituation, and the Morris water maze (MWM) after exposure to 0.5 and 1.0 mg/kg/day of CZP between P7 and P11. The lower part shows a timeline for passive avoidance after exposure to 1.0 mg/kg/day of CZP.

Behavioral tests were performed in a special room with constant temperature (22 ± 2°C) and light conditions (35–45 lx). Before testing, animals were allowed to adapt to the testing room for 30 min. All tests were performed between 9:00 AM and 3:00 PM. The detailed experimental schedule is summarized in Figure 1. The same animals (controls *n* = 12; CZP 0.5 mg/kg/day, *n* = 12; CZP 1.0 mg/kg/day, *n* = 16) were used for the homing test, the habituation test and the MWM test, and the order of tests was always the same (homing test → habituation test → MWM). An additional 120 animals were used for the passive avoidance test. For each age group tested (P12, P15, P18, P25, P32), naïve animals were always used (controls *n* = 12; CZP 1.0 mg/kg/day, *n* = 12). The control group and the group exposed to CZP at the dose of 1.0 mg/kg each consisted of 12 animals. Behaviors in the OF and on the MWM were video-recorded and then analyzed using EthoVision (Noldus Information Technology).

### Homing response (HR) test

The homing response (HR) procedure exploits the motivation of a rat pup to reach its home nest and maintain contact with its dam and siblings. This test is suitable and biologically relevant for examining the spatial learning in immature rodents as remembering the home localization has a high adaptive value in pre-weaning animals. Cooperation between olfactory and/or visual cues is considered to be an important factor in mastering the HR (Rossier and Schenk, 2003; Mikulecká and Mareš, 2009). The same procedure as in our previous work was employed (Mikulecká and Mareš, 2009). The same groups of animals were tested repeatedly at four different ages: P12, P15, P18, and P23. The following parameters were quantified: (1) mean latency to homing; (2) ratio of correct responses (latency < 60 s) to the total number of trials (correct/total responses × 100); and (3) occurrence of five consecutive correct HR (HR acquisition). The number of animals that fulfill these criteria was calculated.

### Passive avoidance responding

The immature and juvenile animals were subjected to a three-trial step-through passive-avoidance paradigm (Carey et al., 2001). Animals (naive groups for each age group) were tested at P12, P15, P18, P25, and P32. The apparatus (Ugo Basile, Italy) was a rectangular Plexiglas cage (47 × 18 × 26) for P12, P15, and P18, and (52 × 30 × 35 cm) for P25 and P32 rats, consisting of two compartments of equal size. The animals were tested in three trials (at 0, 2 and 24 h intervals). The 2nd and the 3rd trial were performed without delivery any shock to measure short-term memory (STM) and LTM retention, respectively (Izquierdo et al., 1999).

### Open field test (OF)

Within- and between-session habituations were evaluated in P70 to P73 animals using the method described and validated by Thiel and collaborators in the OF test (Thiel et al., 1999). The OF arena consisted of a square black plastic box (48 × 48 cm, walls 30 cm). Animals were tested for 4 consecutive days (one 10 min session each day). The following behavioral variables were analyzed automatically. Locomotion (distance moved) was evaluated by analyzing the track record for the distance traveled and center time (i.e., time in the central 30 × 30 cm section of the OF). Habituation of locomotion was evaluated in two ways. Within-session habituation was measured by comparing the 1st vs. the 2nd 5 min interval of a given testing period (session). As previous studies demonstrated that between-session habituation could most appropriately be described by comparing the 1st session with the 4th session of 4 day consecutive exposure to the OF (Thiel et al., 1999), a comparison between the 1st day (1st session) vs. the 4th day (4th session) was used for the data analysis and presentation.

### Morris water maze (MWM)

Place learning and long-term spatial memory were tested in adult animals (P80–P84) using the MWM (Morris, 1981, 1984; D’Hooge and De Deyn, 2001). The MWM consisted of a black circular pool (210 × 50 cm) filled with clear water (20°C). A circular transparent Plexiglas platform (10 cm in diameter) was submerged 1.5 cm below the surface of the water in the center of an arbitrarily defined quadrant of the pool (northwest) and remained in the same position throughout the testing. In the acquisition test, each rat received one session (8 trials) per day for 5 consecutive days. A trial began by placing the animal into one of the four psuedo-random starting positions (N, W, S, or E). In case the rat failed to locate the platform in 60 s, the experimenter guided the rat to the platform where it was allowed to rest for 30 s. After each session, the rat was dried with a towel and kept in a warmed cage. Escape latency (time to reach the hidden platform) was measured. To test the rat’s knowledge of the hidden platform location, a spatial probe trial was run immediately after the completion of the 5th session. The platform was then removed from the maze and rat was allowed to swim freely for 90 s. The time spent in the quadrant that previously contained the platform was measured. A short re-acquisition test (one session) was performed in the same manner as the acquisition, 10 and 40 days after the final acquisition session (at P95 and P125, respectively). Re-acquisition sessions were conducted to determine the ability to retrain as a simple controlled condition compared to acquisition and probe.

## Statistical analysys

Because the data from the HR test did not meet the assumption of equal variance of parametric ANOVA, the Friedman repeated-measures analysis using Student-Newman-Keuls method was applied to compare the individual age groups. The Kruskal-Wallis analysis with *post-hoc* Dunn’s method was applied to compare the individual age groups. The differences in HR acquisition between the control and the CZP groups were evaluated by means of the χ^2^-test. The data set from the passive avoidance, habituation and MWM tests were analyzed by two-way repeated-measure ANOVA with one between-group factor (treatment) and one within-subject factor (repeated session). During the habituation experiment, as a computer error affecting the data collection occurred in two animals, 14 animals were included in the group exposed to CZP at the dose of 1.0 mg/kg for analysis. The *post-hoc* Holm-Sidak method was used to explore main significant effects or interactions resulting from ANOVA. The level of significance was set at *P* < 0.05 (Sigma Stat®, SPSS Inc., Chicago, IL). To simplify the text, only statistically significant values are provided in the Results section.

## Results

### Body weight

**Figure 2:**
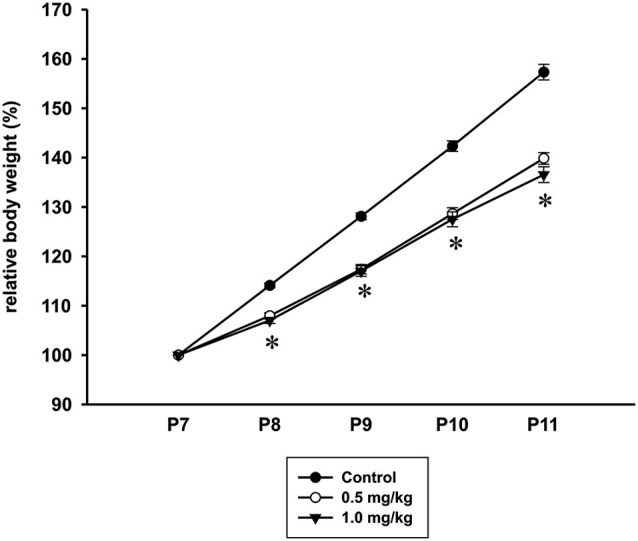
**Body weight (mean + SEM) of rats during CZP exposure**. Abscissa: age in days. Ordinate: relative weight (body weight was measured on the 1st day of drug administration, 100%). * Significant difference compared to appropriate control group.

The control animals gained more weight than the CZP-exposed rats during CZP administration. From P8 to P11, the relative body weight was significantly lower in animals treated with CZP at both doses (0.5 and 1.0 mg/kg) than in the controls [drug effect: *F*_(2,148)_ = 35.7, *p* < 0.001; age *F*_(4,148)_ = 1450.8, *p* < 0.001; drug × age interaction *F*_(8,148)_ = 22.8, *p* < 0.001] (Figure 2).

### Homing response (HR) test

**Figure 3:**
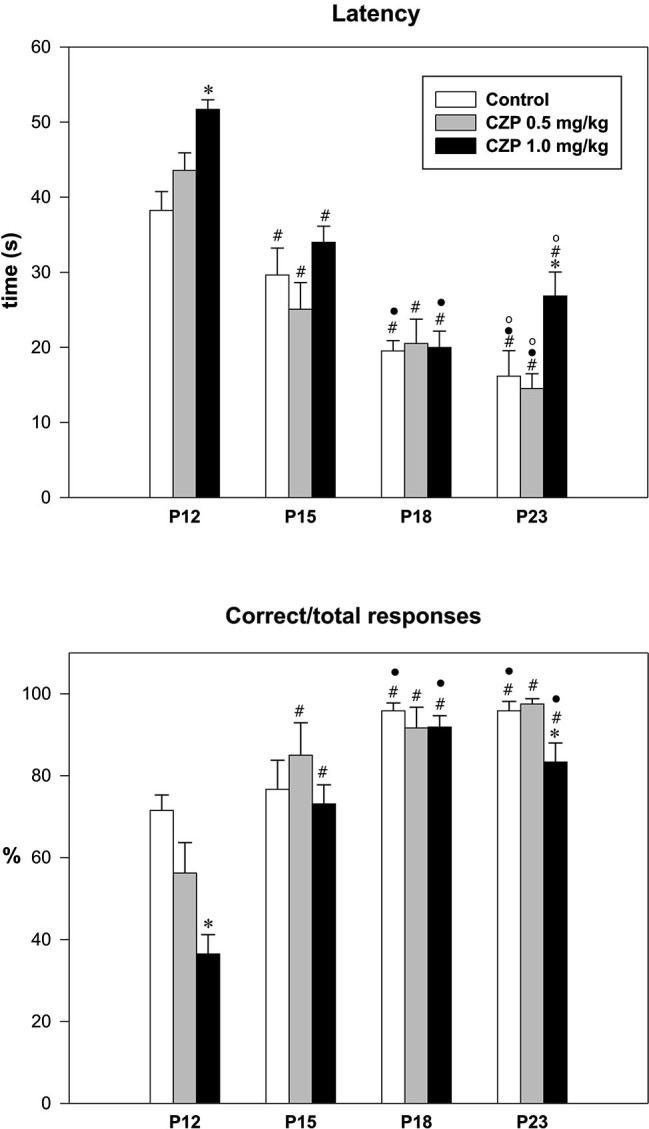
**Effects of CZP exposure on HR**. Upper graph—latency to reach home part of the cage; lower graph—ratio of correct to total responses. Abscissae: four age groups. Ordinate for the upper graph: time in seconds. Ordinate for the lower graph: percentage of correct responses. For individual doses, see inset. * Significant difference compared to an appropriate control group, ^#^ significant difference compared to P12 animals, • significant difference compared to P15 and º significant difference compared to P18 animals.

HR improved gradually during development. In the controls, homing latency decreased and the ratio of correct responses increased gradually with age (*p* < 0.001), while exposure to CZP at either dose did not affect this developmental trend (*p* < 0.001). In P12 rats, CZP at the dose of 1.0 mg/kg increased the homing latency and decreased the ratio of correct responses (*H* = 16.4, *p* < 0.001 and *H* = 15.8, *p* < 0.001, respectively). No significant differences were found in either evaluated parameters at P15 or P18. In P23, rats the high CZP dose (1.0 mg/kg) prolonged the homing latency and decreased the ratio of correct responses (*H =* 9.6*, p =* 0.008 and *H =* 8.2*, p =* 0.01, respectively). In controls, the HR learning improved with age. The criteria for correct HRs were achieved in 75% of the P12 and P15 rats, and in 100% of the P18 and P23 animals. Conversely, the CZP worsened the HR in a dose and age dependent manner. In P12, only 50% treated with 0.5 mg/kg and only 6.3% treated with 1.0 mg/kg learned the HR. In P15 rats, 91.6% rats treated with 0.5 mg/kg and 75% treated with 1.0 mg/kg achieved the criteria for the HR. In P18 and P23 rats, all animals treated with the 0.5 mg/kg dose achieved the criteria for correct HRs. In P23, only 56.3% rats treated with the high CZP dose were able to achieve the criteria for five consecutive correct responses (Figure 3).

### Passive avoidance responding

**Figure 4:**
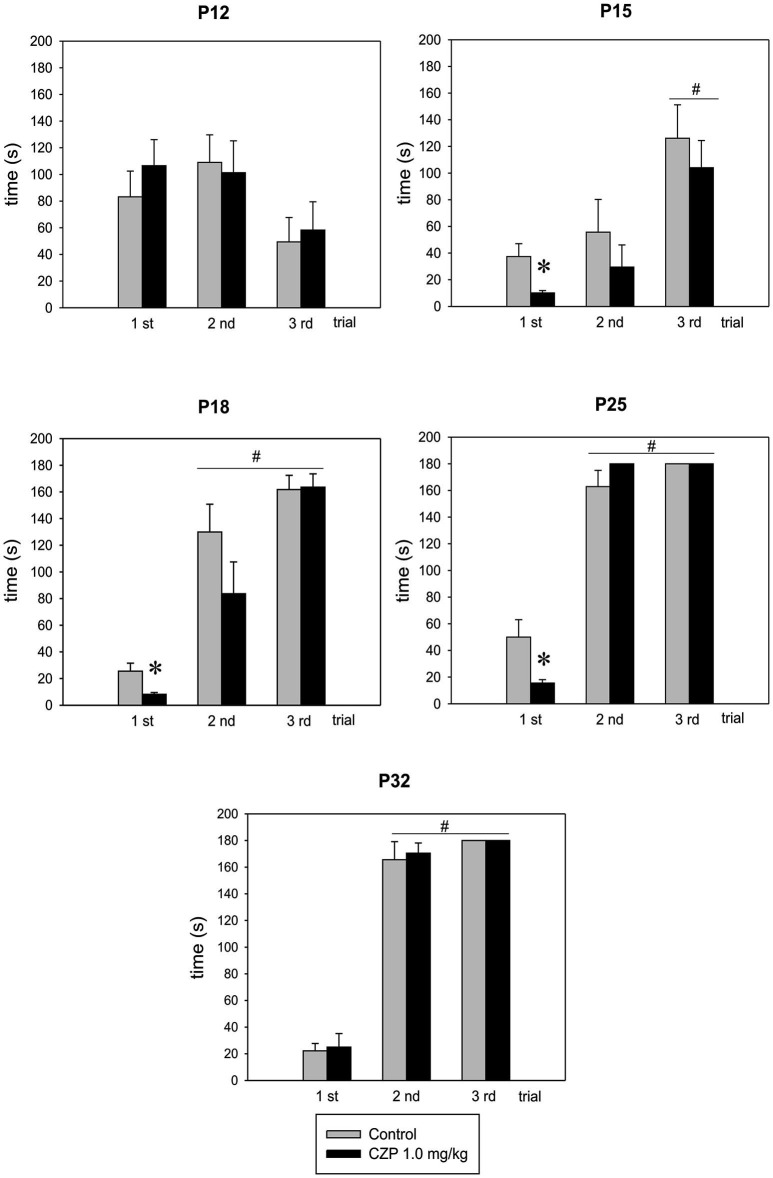
**Effect of CZP exposure (1.0 mg/kg) on passive avoidance retention performance**. The animals were tested at P12, P15, P18, P25 and at P32. Abscissae: the 1st, the 2nd and the 3rd trials. Ordinate: time of entry latencies in seconds. * Significant differences compared to an appropriate control group; ^#^ significant difference between sessions.

In the first trial, step-through latency decreased with maturation. In the retention trials, performed at 2 h and at 24 h, the latency increased continuously as a function of age, indicating the development of avoidance memory. CZP did not affect this developmental trend (*p* < 0.001 for both controls and CZP-exposed animals). Criteria set for full retention was achieved at P25 in both the controls and the CZP-exposed animals (*p* < 0.001). Passive avoidance response was not present in P12 rats in any trial, and CZP had no effect. In the first trial, the CZP-exposed animals tested at P15, P18, and P25 displayed shorter step-through latencies than the controls. This suggested that all CZP animals reacted differently than the controls to a new environment, with low exploration of the light box and suppressed risk-assessment behavior (animals enter the dark box without hesitation), whereas control animals demonstrated caution and risk-assessment behavior before walking into the dark box. This behavior was not observed in P32 animals. In P15, the step-through latency increase was observed only at 24 h after the first trial in both control and CZP-exposed rats [*F*_(2.44)_ = 21.9, *p* < 0.001]. Starting at P18, the step-through latency significantly increased in both retention trials, that is, 2 and 24 h after the first trial [*F*_(2,44)_ = 55.9, *p* < 0.001; *F*_(2,44)_ = 289.4, *p* < 0.001; *F*_(2,44)_ = 342.2, *p* < 0.001, respectively]. There was no difference between the controls and the CZP animals, suggesting that CZP exposure did not impair the retention of memory avoidance (Figure 4).

### Open field (OF)

**Figure 5:**
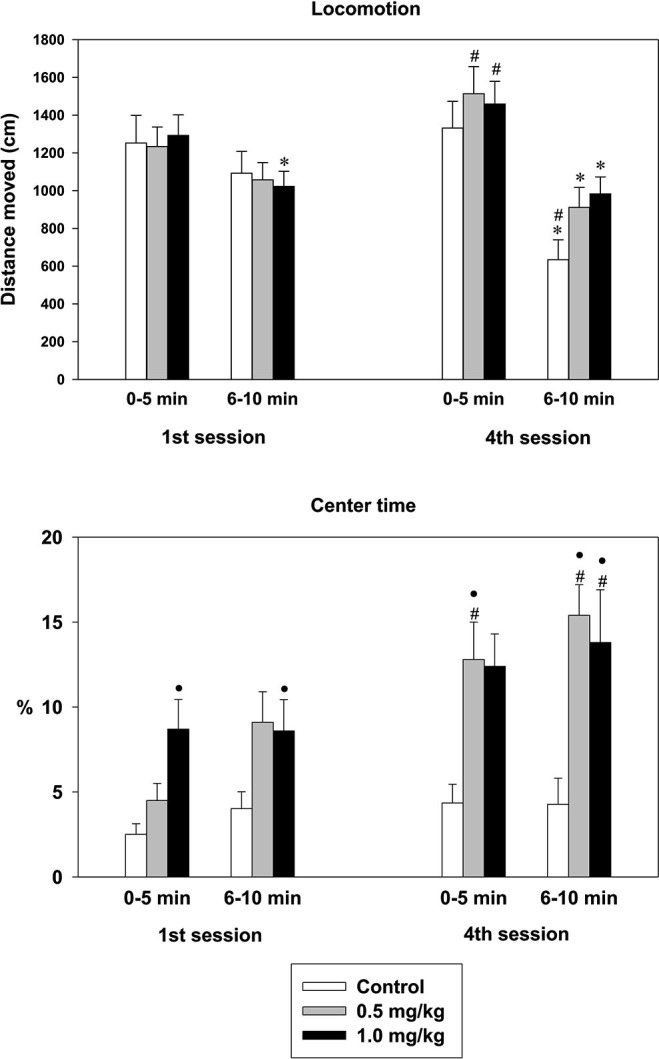
**Effect of CZP exposure on habituation in adult animals**. Upper graph—locomotor activity measured as the distance moved in the open field. Abscissae: between-session habituation (1st session vs. 2nd session) and within-session habituation (1st session vs. 2nd session, 5 min interval for a given testing period). Ordinate: distance moved in cm. * Significant difference in within-session habituation compared to an appropriate group. ^#^ Significant difference in between-session habituation. Lower graph: time spent in the center part of the OF. Abscissae: similar to upper graph. Ordinate: time expressed in percentage. • Significant difference compared to controls.

In the 1st session, distance moved decreases with time in both the control and the CZP-exposed animals [*F*_(1,35)_ = 9.6, *p* = 0.004]. A comparison between the 1st 5 min and 2nd 5 min interval revealed significant difference only in the 1.0 mg/kg. CZP-exposed animals In the 4th session, both the control and the CZP-exposed animals showed significant decreases in distance moved, which was more expressed within-session habituation with repeated sessions [*F*_(1,35)_ = 47.1, *p* < 0.001], thus indicating that the animals adapted to the experimental conditions. As for between-session habituation, the comparison of the 1st 5 min interval of a given session showed significant increases in distance moved in CZP-exposed animals with repeated exposure [*F*_(1,35)_ = 4.8, *p* = 0.03]. Furthermore, the *post-hoc* test revealed significant increases in the distance moved in animals exposed to both doses of CZP in the first 5 min interval of the 4th session, suggesting a lack of between-session habituation in the 1st 5 min interval. A comparison of the 2nd 5 min interval of a given session revealed significant decreases in distance moved with repeated sessions [*F*_(1,35)_ = 12.1, *p* = 0.001]. However, the *post-hoc* test confirmed decreases in the distance moved only in the control animals, thus suggesting between-session habituation in the controls, but not in the CZP-exposed animals. In addition, the distance moved in the 2nd 5 min interval was significantly shorter in controls than in the CZP-exposed animals, thus indicating hyperactivity of the treated rats (Figure 5). Animals exposed to CZP in either dose tended to increase the time spent in the central zone of the OF. In the 1st session, this tendency was dose- and time-dependent [*F*_(2,35)_ = 4.1, *p* = 0. 02; *F*_(1,35)_ = 4.6, *p* = 0.03, respectively], and in both 5 min intervals, it reached the level of significance only in animals exposed to CZP at the dose of 1.0 mg/kg. A similar dose- and time-dependent trend was observed in the 4th session [*F*_(2,35)_ = 6.3, *p* = 0.005;* F*_(1,35)_ = 10.04, *p* = 0.003, respectively]. CZP increased the center time in the 4th session compared to the 1st session in both 5 min intervals. For the 1st interval, there were significant main effects of treatment [*F*_(2,35)_ = 0.7, *p* < 0.001] and session [*F*_(1,35)_ = 11.8, *p* = 0.001]. Similarly, there were significant main effects of treatment [*F*_(2,35)_ = 7.6, *p* = 0.002] and session [*F*_(1,35)_ = 5.07, *p* = 0.03] for the 2nd interval, thus indicating hyperactivity/excitability of CZP-exposed animals (Figure 5).

### Morris water maze (MWM)

**Figure 6:**
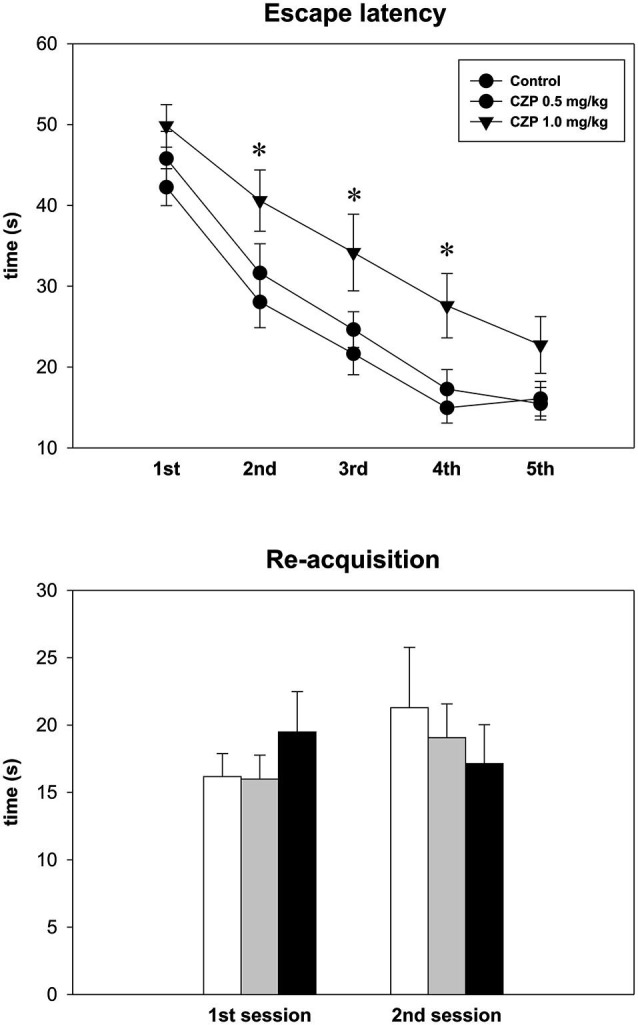
**Effect of CZP exposure on long-term memory (LTM) version of the Morris water maze (MWM)**. Upper graph: escape latencies per daily sessions (each session consists of 8 trials). Lower graph: re-acquisition test. Abscissae: 1st session (10 days after the final acquisition session) and 2nd session (40 days after the final acquisition session). Ordinate: escape latencies (s). * Significant differences compared to controls in the appropriate session.

In both the control and the CZP-exposed animals, the time required to locate the platform decreased over the five successive sessions [*F*_(4,148)_ = 82.4, *p* < 0.001]. Furthermore, there was no difference between rats exposed to the lower dose of CZP (0.5 mg/kg) and the controls. In contrast, rats exposed to CZP at the dose of 1.0 mg/kg had significantly longer latencies in the 2nd, 3rd, and 4th sessions [*F*_(2,148)_ = 4.5, *p* < 0.01]. In the probe trial, which immediately followed the final acquisition session, the controls and the animals exposed to CZP at the dose of 0.5 mg/kg spent more time in the quadrant where the platform was placed during training (50.8%, 49.3% of 90 s, respectively) compared to the animals exposed to the 1.0 mg/kg dose of CZP (36.4%) [*F*_(2,35)_ = 5.0, *p* = 0.01]. On the contrary, no difference was found between the 5th session of the acquisition test and the 1st re-acquisition test for both the control and the CZP-treated rats. In addition, there was no difference between the 1st and the 2nd re-acquisition sessions. These data may suggest that in the reference memory version of the MWM, the high dose of CZP impaired acquisition and memory strength in the probe, but did not affect the re-acquisition of a previously pre-trained skill in a familiar environment with the same goal position (Figure 6). The lower dose was without effect in this test.

## Discussion

Results of the present study indicate that even short lasting exposure of postnatal rats to CZP in an anticonvulsant dose range leads to mild alterations of behavioral responsiveness in cognitive-related behavior in immature, juvenile and adult animals. While developing animals were exposed to CZP for only 5 days, the exposure occurred during the brain growth spurt when there is considerable brain plasticity that is critical for network formation. The newborn rat is equivalent to a 6-month-old human fetus, and P12 rats are equivalent to early infancy in humans (Dobbing and Sands, 1979; Avishai-Eliner et al., 2002; Clancy et al., 2007). Therefore, we assume that the level of maturation of the rat brain during the CZP exposure corresponds with the perinatal development of the human brain. Repeated administration of CZP in doses used for our study had only limited effects on body growth, and mortality was negligible. While the drug caused transient growth retardation during the period of administration, the body weight normalized rapidly after P18.

The measurement of cognitive performance in rats incorporates modulatory functions such as sensory, attentional, motivational, emotional, and motor processes (Myhrer, 2003). Immature animals generally show a good learning capacity in conventional or modified tests, which take into account the ethological characteristics and sensorimotor abilities of animals at a particular developmental stage.

In concordance with previously published results (Sigling et al., 2009), our data show a gradual increase in the preference for the home nest from postnatal P12 with a maximal preference at P23. The performance of pups on this test improved during maturation, while the latency to homing decreased and the percentage of correct responses increased with age. This developmental pattern reflects a gradual maturation of rat sensorimotor systems (Altman and Sudarshan, 1975; Spear, 1990). Exposure to CZP did not affect the developmental trend evidenced in the controls. Nevertheless, the administration of CZP at a dose of 1.0 mg resulted in a latency prolongation and a decrease of correct HRs in P12 rats. We previously showed that after a single administration of CZP at P12, motor abilities were compromised for 4 h, whereas anxiety-like behavior was suppressed for 48 h (Mikulecká et al., 2011). These data suggest that higher plasma levels of CZP are necessary to affect motor behavior. While data on the pharmacokinetics of CZP in rats are sparse (Hoogerkamp et al., 1996) showed that the terminal half-life of CZP in adult rats is approximately 1 h. Given that immature organisms eliminate CZP more slowly than adults (Markowitz et al., 2010), we speculate that factors other than the sedative effects of CZP are responsible for the alteration of the HR in P12 animals. Interestingly, home-response impairment was also observed in P23 animals, the oldest group tested. Exposed animals preferred the exploration of the empty box. The response delay could be related to decreased motivation of exposed pups to reach the home box/nest, a factor that may have accounted for the learning curve shift. Despite the fact that performance in the HR test does not require place navigation in the narrow sense, i.e., navigation to hidden goals, these results suggest an impairment of learning. Nonetheless, such impairment might also be associated with altered emotionality.

The developmental profile of passive-avoidance responses evidenced in our experiments is consistent with results of previously published studies (Stehouwer and Campbell, 1980). While P12 rats failed to remember the passive-avoidance response, P15 animals were able to recall the association between the context and the foot shock 24 h but not 2 h after the first exposure. These data suggest that long-term memories can be formed by P15, and they support previous findings showing that STM and LTM involve separate mechanisms and that both memory types are independently processed (Izquierdo et al., 1999; Vianna et al., 2000). In accordance with previous studies (File, 1986), our results show that early exposure to CZP does not affect performance in the task requiring a passive-avoidance response. Interestingly, compared to controls, step-through latencies in the first exposure to the apparatus were shorter in P15, P18 and P25 animals exposed to CZP. CZP animals reacted differently to the new environment based on their low degree of exploration of the light box and their suppressed risk-assessment due to their disinhibited response (e.g., excitement) to novelty. Control animals demonstrated caution and risk-assessment behavior before stepping into the dark box. This can be interpreted as a result of differences in the variables of emotional domain and strategies in coping with a task requiring passive-avoidance (Morellini and Schachner, 2006).

Habituation is commonly defined as a change in exploratory or locomotor activity over time (within-session) or as a change in exploratory or locomotor activity with repeated exposure (between-session) (for review Leussis and Bolivar, 2006). CZP exposure did not alter the within-session habituation, indicating intact adaptation to novelty in both the controls and in the CZP-exposed animals. In contrast, alteration of between-session habituation evidenced in CZP-exposed animals suggests an impairment of a non-associative form of learning. Alternatively, it is possible that the CZP-treated animals had problems recognizing the context upon repeated exposure to it, which might be in accord with a pattern found in the MWM (see next paragraph). CZP-exposed animals exhibited higher locomotion in the 4th session and spent more time in the central area of the OF, indicating behavioral disinhibition (hyperactivity/excitability) triggered by novelty and/or decreased anxiety. These data support a delay of habituation described previously in animals exposed to BZDs during gestation (Laviola et al., 1992) or during gestation and lactation (Marczynski et al., 1988). This suggests that, together with altered anxiety, developmental CZP exposure also results in corrupted habituation or context recognition.

Only early exposure to a high dose of CZP led to impairment of learning abilities in the MWM. During the acquisition phase of the test, while escape latencies were longer in the animals of the exposed group, the shape of the learning curve did not differ from other groups. We hypothesize that CZP-exposed animals used a combination of visual and egocentric strategies to localize the platform. Perhaps changes in the strategy, together with a cognitive impairment, are responsible for slower learning (Moghaddam and Bureš, 1996). In addition, in the immediate spatial probe trial (without platform), animals exposed to 1.0 mg/kg of CZP spent a shorter time in the target quadrant, suggesting either impaired strategy or memory trace formation. In contrast, an earlier study (Wang and Huang, 1990) found no learning impairment on the MWM in infant mice exposed to CZP. This difference is likely explained, however, by different experimental procedures used in the study. Nonetheless, we propose that the impairment of probe trial performance in the CZP-treated rats (shorter time in the target quadrant) clearly demonstrates impaired spatial representation in the group treated with the higher dose of CZP.

Taken together, the results of the present study show that even short-term exposure to BZDs during the early postnatal period can result in complex behavioral changes detectable later in life as behavioral alterations had already manifested in pre-weaning animals. These changes were observed both in a domain of emotionality (e.g., time in the OF center) and in measures of learning and memory (e.g., impaired probe trial). Despite the fact that the tests used in the present study do not allow for a clear dissociation between altered affect and cognition, they do suggest that both domains were impaired concurrently. Our results are in concordance with previously published data that demonstrate that changes induced by exposure to BZDs during gestation and infancy are related to changes in emotionality, to changes in levels of hyperactivity/hyperarousal and attention deficit (Frieder et al., 1984; Livezey et al., 1986; Schroeder et al., 1997) and to changes in memory function. This underscores the usefulness of ontogenetic CZP treatment as an animal model of neurodevelopmental disturbance in the GABAergic system, which may manifest in the domain of behavior.

In conclusion, the results of our study demonstrate that in immature rats, even brief exposure to BZDs leads to mild impairment of cognitive abilities and also changes emotional/motivational responsiveness, hyperactivity and attention deficits later in life. Some of these alterations can persist until adulthood. Finally, these results have clinical importance as CZP treatment is routinely used in epileptic children.

## Conflict of interest statement

The authors declare that the research was conducted in the absence of any commercial or financial relationships that could be construed as a potential conflict of interest.
